# Otilonium bromide exhibits novel antifungal activity against *Candida albicans* via regulating iron homeostasis

**DOI:** 10.1080/21505594.2025.2609407

**Published:** 2025-12-23

**Authors:** Li-Hang Hsu, Yuk-Ping Chou, Tang-Long Shen, Daria Wieczorek, Ying-Lien Chen

**Affiliations:** a Department of Plant Pathology and Microbiology, National Taiwan University, Taipei, Taiwan; b Department of Technology and Instrumental Analysis, Poznan University of Economics and Business, Poznan, Poland

**Keywords:** Otilonium bromide, *C. albicans*, repurposing, antifungal drugs, biofilm formation

## Abstract

Traditional antifungal drugs used against *Candida albicans* have several drawbacks, including the emergence of drug-resistant strains. In addition, developing novel antifungal agents requires long-term research and design. Drug repurposing, identifying and utilizing previously unknown functions of known drugs, such as antifungal activity, may be a quick method for mining efficient alternatives. Otilonium bromide (OB), an FDA-approved drug, is a quaternary ammonium compound used as a therapeutic drug for irritable bowel syndrome. We previously reported the inhibitory effect of OB against the spore germination of *Cryptococcus neoformans*. In this study, we found that the antifungal activity of OB against *C. albicans* was 2 μg/mL for both minimum inhibitory and fungicidal concentrations. OB could destroy the cell membrane and prevent *C. albicans* from undergoing yeast-to-hyphae transition, thus interfering with biofilm formation. Additionally, the efficacy of OB was abolished when iron ions were provided, suggesting that iron homeostasis was associated with the inhibition mechanism of OB. Interestingly, a therapeutic assay showed that OB demonstrated limited efficacy in reducing *C. albicans* burden in a murine systemic infection model. In summary, repurposing OB against *C. albicans* may facilitate the design of new antifungal drugs, and chemical modification could enhance the efficacy of OB to be more specific to fungal pathogens.

## Introduction

*Candida albicans* is a commensal yeast found in 70% of healthy humans, especially on the skin and mucosal surfaces [1]. However, being an opportunistic pathogen, *C. albicans* is the dominant species that causes candidiasis in immunocompromised individuals, and it turns into candidemia if not treated appropriately [2]. Nowadays, antifungal drugs are categorized into three classes: azoles, echinocandins, and polyenes. Although the antifungal activity is significant, drug-resistant strains still evolve and survive from drug application [3]. In addition, due to high nephrotoxicity in some drugs and the increased cost of developing new antifungal drugs, alternative approaches for discovering antifungal agents are needed.

Traditional development of new antifungal drugs requires substantial time and cost investments, leading to increased interest in drug repurposing as an alternative strategy. Our previous screening of 1018 FDA-approved drugs identified otilonium bromide (OB), which demonstrated significant inhibitory effects against *Cryptococcus neoformans* germination [4]. This finding aligns with growing evidence that quaternary ammonium compounds (QACs) possess antimicrobial properties beyond their traditional applications. Several QACs have shown antifungal efficacy: benzalkonium chloride exhibits activity against *Candida* species through membrane disruption, while cetylpyridinium chloride demonstrates broad-spectrum antifungal effects [5,6]. Notably, our work with the QAC compound PMT12-BF4 revealed its antifungal mechanism involves interference with iron homeostasis [7], suggesting this pathway may be a common target for QAC-based antifungal activity.

Among the repurposed candidates, OB, an FDA-approved drug, demonstrated inhibitory activity on yeast germination in *Cryptococcus neoformans*, and the bacterial pathogen *Acinetobacter baumannii* [4,8]. OB is a quaternary ammonium compound (QAC) used to treat irritable bowel syndrome (IBS). Patients suffering from IBS have abnormal contraction of the intestinal tract, followed by cramps in the abdomen. In terms of its mechanism in mammals, OB inhibits the nerve impulse by blocking calcium ion channels and tachykinin receptors. Thus, those ligands are not accessible to the intestine, interrupting signal transduction and alleviating patient pain [9–11].

Trace elements such as calcium, iron, copper, or zinc are essential for the growth of most organisms. Among all the trace elements, iron is most closely associated with the survival and virulence of *C. albicans* in the host environment [12]. Iron sequestering thus plays an important role in humans to prevent *C. albicans* from invading, which is called nutritional immunity [12,13].

In this study, we focused on OB and its antifungal activity and found a potential pathway associated with iron homeostasis that inhibits the growth of *C. albicans*. Understanding this mechanism could provide valuable insights for developing more effective antifungal strategies through drug repurposing, particularly targeting iron homeostasis as a novel therapeutic approach.

## Materials and methods

### Strains, compounds, and media

The strains used in this study are listed in Table 1. OB (MedChemExpress, USA) as a pure compound was dissolved in dimethyl sulfoxide (DMSO, Scharlab, Spain) to make a 5 mg/mL stock solution for further experiments. Fluconazole (Pfizer, USA) in the form of 2 mg/mL and Catilon tablets containing 40 mg of OB per tablet (kindly provided by Tri-Service General Hospital, Taiwan) was used for the murine therapeutic assay.

**Table 1.** Minimal inhibitory concentration (MIC) and minimal fungicidal concentration (MFC) of otilonium bromide against multiple human fungal pathogens.

Strains (reference)	Description	MIC	MFC
*Candida albicans* SC5314 [14]	Wild type	2	2
*Candida albicans* 12–99 [15]	Fluconazole resistant	2	2
*Candida albicans* 89 [16]	Echinocandin resistant	2	2
*Candida albicans* Δ*cna1*/Δ*cna1* [17]	Calcineurin mutant	2	2
*Candida albicans* Δ*cnb1*/Δ*cnb1* [18]	Calcineurin mutant	2	2
*Candida dubliniensis* CD36 [19]	Wild type	2	2
*Candida tropicalis* MYA3404 [20]	Wild type	2	2
*Candida tropicalis* DPL73 [21]	Echinocandin resistant	1	1
*Candida parapsilosis* J941367 [20]	Wild type	1	1
*Candida glabrata* CBS138 [22]	Wild type	8	8
*Candida glabrata* DPL23 [23]	Echinocandin resistant	4	8
*Candida glabrata* AmB-resistant isolate D [24]	Amphotericin B resistant	4	8
*Cryptococcus neoformans* H99 [25]	Wild type	4	4
*Cryptococcus neoformans* T1 [26]	Fluconazole resistant	2	2
*Cryptococcus neoformans* 89–610 [26]	Fluconazole resistant	2	2
*Cryptococcus deuterogattii* R265 [27]	Wild type	2	2
*Aspergillus fumigatus* AF293 [28]	Wild type	4	4
*Aspergillus fumigatus* A031 [29]	Azole resistant	32	32

Media used in this study were YPD (1% yeast extract [Bioshop, Canada], 2% peptone [Bioshop], 2% dextrose [Bioshop]), PDB (24 g potato dextrose broth [Himedia, India] in 1 L distilled water), LB (0.5% yeast extract, 1% tryptone [Bioshop], 1% NaCl), RPMI 1640 (10.4 g RPMI 1640 powder [Sigma-Aldrich, USA], 34.5 g MOPS [3-(N-morpholino) propanesulfonic acid, Sigma-Aldrich], 2% dextrose, in 1 L distilled water, with pH adjusted to 7.0 with NaOH), Spider medium (10 g nutrient broth [Himedia], 10 g mannitol [Pancreac, Spain], 2 g K_2_HPO_4_, in 1 L distilled water, adjusted to pH 7.2 with H_3_PO_4_), SLAD (synthetic low ammonium dextrose, 0.17% yeast nitrogen base without amino acids or ammonium sulfate [Bioshop], 50 μM (NH_4_)_2_SO_4_, 2% dextrose), and YNB (0.17% yeast nitrogen base without amino acids or ammonium sulfate, 0.5% (NH_4_)_2_SO_4_, 2% dextrose). All media were solidified by adding 2% agar (Bioshop) if necessary.

### Determination of antifungal activity

The minimal inhibitory concentrations (MICs) were obtained following the CLSI protocol M27-A3 and M38-A2. For yeasts, strains were cultured in 3 mL YPD broth medium overnight at 30°C, washed twice, and resuspended with ddH_2_O, and the concentration was determined using a hemocytometer. For *Aspergillus fumigatus*, strains were streaked out on a PDA agar plate and incubated for 5 days at 37°C. Spores were collected with sterile water, filtered through Miracloth (Millipore, USA) to remove hyphae, and the concentration was determined by hemocytometer. All inoculums were then diluted to the recommended concentrations with RPMI 1640 medium. OB was serially diluted 2-fold by RPMI 1640 medium, and 100 μL of the compound was added to a 96-well plate, followed by 100 μL of the inoculums. The final concentration of OB ranged from 0.125 - 64 μg/mL, and the final concentrations of the inoculums were 1.25 × 10^3^ cells/mL for yeasts and 2.5 × 10^4^ for *A. fumigatus*. Plates were incubated at 35°C for 48 h, and the MIC was defined as the lowest concentration at which no visible colony grew. Minimal fungicidal concentration (MFC) was determined after the MIC test. For each strain, 3 μL of wells containing OB at concentrations from 0.5 MIC (positive control) to the highest concentration (64 μg/mL) were thoroughly pipetted and subcultured onto drug-free YPD or PDA agar plates. The plates were incubated at an optimal temperature for growth of each fungal pathogen for 48 h and the MFC was determined as the drug concentration at which no colonies formed.

### Time-kill kinetic assay

Overnight cultures of *C. albicans* SC5314 were washed twice with ddH_2_O and inoculated into YPD broth to a volume of 3 mL (10^5^ CFU/mL) with or without 8 μg/mL OB at 30°C. A 100 μL aliquot was removed from each culture at the indicated time points (0, 4, 8, 12, 24, and 48 h), appropriately diluted with ddH_2_O, plated on a fresh YPD agar plate and incubated at 30°C for 48 h before colony count. Two-way ANOVA was performed using GraphPad Prism 6, and a *p* value of less than 0.05 represented a significant difference.

### Biofilm formation assay

Overnight cultures of *C. albicans* SC5314 were washed twice with PBS and diluted with 2 mL Spider medium to 0.5 OD_600_. OB was added at the indicated concentrations after 1 h incubation at 37°C for cell adhesion. After incubation for 48 h, the medium and suspended cells were removed by pipetting; the biofilm was washed with PBS twice, stained with 0.4% crystal violet and destained with 99% EtOH. The OD_595_ was measured after the destaining step, and wells without OB were defined as having 100% biofilm formation. Unpaired t-tests were performed using GraphPad Prism 6, and a *p* value of less than 0.05 represented a significant difference.

### Hyphae induction and membrane penetration assay

For hyphal induction assay, *C. albicans* SC5314 cells cultured in YPD broth overnight at 30°C were washed twice with ddH_2_O and diluted with 2 mL RPMI 1640 medium to OD_600_ 0.25 in a 12-well plate (Nest Biotechnology, China). OB was added into wells at the concentrations of 0, 1, 2, or 4 μg/mL, with three replicates. The plate was incubated at 37°C for 3 h for germ tube induction and observed with an inverted microscope (Olympus, Japan) at 400X magnification. Cells were photographed using a camera connected to Olympus cellSens Entry 2.1 software. In addition, cells were also adjusted to 10^3^ cells/mL with ddH_2_O and 50 μL of cells were plated on solid Spider or SLAD medium with 0, 5, or 10 μg/mL OB. After incubation for 24 h at 37°C, colonies were observed using a microscope at 40X.

For membrane penetration assay, the induced germ tube was washed with ddH_2_O, stained with 1 μg/mL propidium iodide, and observed with a fluorescence microscope (Olympus BX53) at 1000X magnification.

### RNA-sequencing experiments

RNA extraction was performed following the previous study [7]. In brief, overnight cultures of *C. albicans* SC5314 cells were washed twice with sterile water, and adjusted to OD_600_ 0.25 with 5 mL fresh YPD in the presence or absence of 0.5 μg/mL OB. The cultures were incubated at 30°C for 3 h with shaking at 200 rpm before RNA extraction. TRIzol reagent (Invitrogen, USA) was used in total RNA extraction following the manufacturer’s protocol. The 1 mL TRIzol was added to collected cells and alternately vortexed for 30 s with 2 mm beads and put on ice for 30 s five times. After being centrifuged at 4°C/12,000 g for 10 min, the supernatant was transferred to a new tube and incubated for 5 min at room temperature. The 200 μL of chloroform was then added and mixed thoroughly, and tubes were incubated at room temperature for 3 min, centrifuged at 4°C/12,000 g for 15 min. The upper layer of supernatant was separated to another tube, and 400 μL isopropanol was added for RNA precipitation. After incubated at room temperature for 10 min, the tubes were centrifuged at 4°C/12,000 g for 10 min, and the supernatant was discarded. The RNA pellet was washed twice with 75% ethanol and resuspended with RNase-free water.

The Next Generation Sequencing (NGS) library construction using RNA was as described in a previous study [30]. The mRNA was amplified with oligo(dT) magnetic beads and shortened into approximately 200-base fragments. The double-strand cDNAs were synthesized using a random hexamer, dNTPs, buffer, and RNase H (for the first strand), and DNA polymerase I (for the second strand). The double-strand cDNAs were purified, and end preparation and 3′ end single nucleotide adenine addition were performed. Sequencing adaptors were then ligated to the fragments and amplified by PCR. Sample library was qualified and quantified with an Agilent 2100 bioanalyzer and ABI StepOne Plus real-time PCR system. Sequencing of the library was done by an Illumina H-Seq 2000 instrument.

### Real-time qRT-PCR

A Turbo DNA-free kit (Invitrogen, USA) was used in removing genomic DNA, and 1 μg of total RNAs were reverse transcribed to cDNA using a high-capacity cDNA reverse transcription kit (Applied Biosystems, USA). Real-time PCR was then performed in mixtures including 1 μL of cDNA (1 ng), 5 μL of Fast SYBR green master mix (Applied Biosystems), 1 μL of 5 μM forward primer, and 1 μL of 5 μM reverse primer. Primer pairs used in real-time PCR are listed in Supplementary Table S1 (Table S1). Quantitative PCR conditions were set as follows: 95°C/7 min denaturation, 95°C/10 s, and 60°C/30 s (40 cycles) amplification; 95°C/15 s, and then 60 s each at 0.3°C increments between 60°C and 95°C (for melting curve). Cycle threshold (C_*T*_) values were obtained using a StepOnePlus system and StepOne software (v.2.3), and the relative gene expressions (2^*-ΔΔCT*^) were calculated based on *ACT1*-calibrated values. The relative expression levels of *C. albicans* genes in the presence of 0.5 μg/mL OB were normalized to those in the absence of OB, and the diagrams were depicted by GraphPad Prism 6.0 software. Significant differences were analyzed using an unpaired *t* test (*p* < 0.05).

### Spot plating assay

*C. albicans* SC5314, and two drug-resistant *C. albicans* 12–99 and 89 isolates were incubated in 30°C overnight, washed twice and adjusted to 0.2 OD_600_ with ddH_2_O. Three microliters of cell suspensions in 5-fold serial dilution were transferred to YNB agar plates containing OB (0, 12.5, or 15 μg/mL) or FeSO_4_ (0 or 10 μg/mL). Plates were incubated at 30°C for 48 h and then photographed.

### Therapeutic assay

For inoculum preparation, cells of *C. albicans* SC5314 were grown overnight in liquid YPD medium, washed twice with phosphate-buffered saline (PBS), and adjusted to a concentration of 5 × 10^6^ cells/mL with PBS. Five- to six-week-old male ICR mice (BioLasco Taiwan, Taiwan) were inoculated with 10^6^ *C. albicans* cells in 200 μL by tail-vein injection. For oral administration, OB was dissolved in a solvent containing 50% Kolliphor (Sigma-Aldrich, USA) in absolute ethanol (Bioman Scientific, Taiwan), and 10-fold diluted with PBS. Then, OB or fluconazole (FLC) was administered orally to mice at the indicated concentrations at 4, 24, 48, 72 h after the inoculation. Ten mice per group were used, and all mice were monitored for 10 days. The mice which lost 20% weight or more after inoculation were euthanized and counted deaths. For euthanasia, the mice were placed in a sealed chamber, and carbon dioxide was introduced (flow rate: 10% − 30% volume/min; flowmeter reading between 1 and 2 liters per minute). Once the animal exhibited no apparent signs of life, the chamber was opened to allow carbon dioxide to dissipate for approximately 2 minutes, after which cervical dislocation was performed as a secondary method to ensure euthanasia. The euthanasia procedures were carried out in accordance with the American Veterinary Medical Association. All mice were included in the experiment, and data were statistically analyzed using a log-rank test using GraphPad Prism 6. All animal experiments were conducted in the Animal Resource Center at National Taiwan University (NTU), and adhered to the ARRIVE guidelines.

## Results

### Otilonium bromide possessed broad-spectrum antifungal activity

In our previous study, a collection of 1018 FDA-approved drugs was screened and assessed for anticryptococcal activity [4]. Among these drugs, OB was able to inhibit *Cryptococcus neoformans* and *Cryptococcus deuterogattii*, and we found that it also inhibited *Candida* species. *C. albicans* strains, including strain SC5314 and drug-resistant isolates 12–99 and 89 were inhibited by OB with MIC at 2 μg/mL. To further investigate whether the antifungal activity of OB involves the calcineurin signaling pathway, we tested its effects on calcineurin-deficient *C. albicans* mutants *Δcna1/Δcna1* and *Δcnb1/Δcnb1*. These mutants are known to exhibit defects in stress responses and cell wall integrity, often resulting in hypersensitivity to various antifungal agents. OB retained antifungal activity against both mutants, suggesting that its mode of action does not require a functional calcineurin pathway. This finding implies that OB likely acts through a mechanism independent of calcineurin signaling. Although similar results were found in *Candida tropicalis*, *Candida glabrata* was more tolerant to OB as the MIC was higher at 8 μg/mL (Table 1). The fungicidal activity of OB was then investigated after the susceptibility test. All tested strains treated with OB at respective MICs were unable to grow when transferred to fresh YPD medium, indicating that OB possessed fungicidal activity and effectively eliminated these pathogens.

### Otilonium bromide inhibited yeast-to-hyphae transition in *C. albicans*

**Figure 1.**
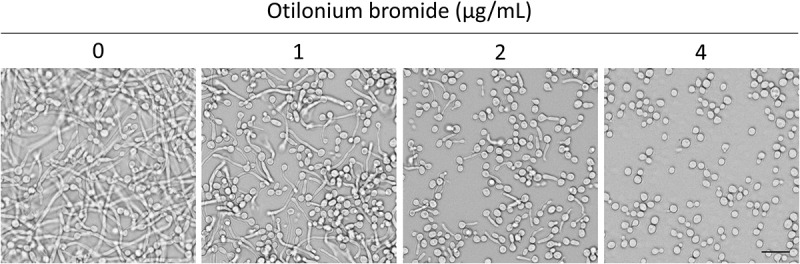
Otilonium bromide inhibited *C. albicans* from germination.

**Figure 2.**
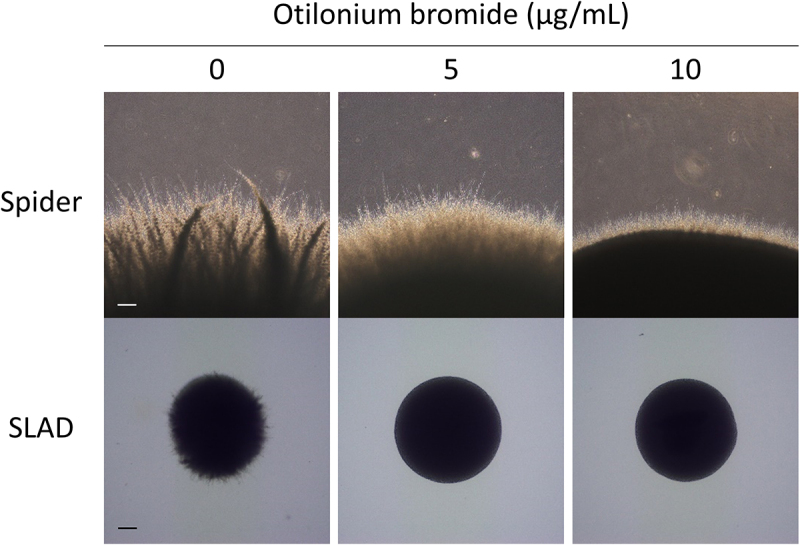
Otilonium bromide inhibited hyphal growth in *C. albicans*.

Yeast-to-hyphae transition is an important virulence factor for *C. albicans* to infect its host; thus we observed whether OB could prevent yeast cells from germination. With exposure to OB for 3 h, few cells were still showing hyphal growth at the concentration of 2 μg/mL OB and lost the ability to germinate and maintain yeast form at 4 μg/mL (Figure 1), suggesting that *C. albicans* were inhibited from germination as the concentration of OB increased. In addition, *C. albicans* was incubated on solid agar medium in hyphal-inducing conditions to evaluate hyphal growth. As the amount of OB increased, the hyphae growing on the edge of the colony became short, suggesting the inhibition of yeast-to-hyphal transition was concentration-dependent (Figure 2).

*C*. *albicans* SC5314 overnight culture was washed twice with ddH_2_O, diluted with 2 mL RPMI 1640 medium to 0.25 OD_600_, and otilonium bromide was added at indicated concentrations. After 3 h incubation at 37°C, cells were observed with an optical microscope at 400X. Scale bar = 20 μm.

*C*. *albicans* SC 5314 overnight culture was washed twice and diluted with ddH_2_O to 100 cells/mL, and 50 μL was spread on Spider or SLAD agar plates with 0, 5, or 10 μg/mL otilonium bromide. After 24 h incubation at 37°C, colonies were observed using a microscope at 40X. Scale bar = 200 μm.

### Otilonium bromide caused membrane damage in *C. albicans*

**Figure 3.**
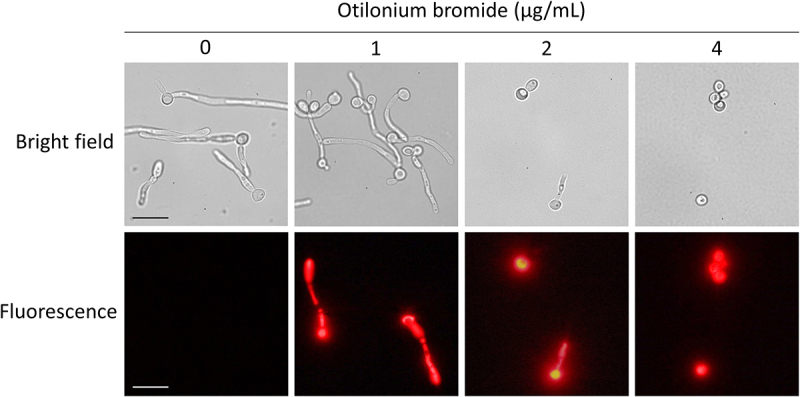
Otilonium bromide caused membrane damage in *C. albicans*.

**Figure 4.**
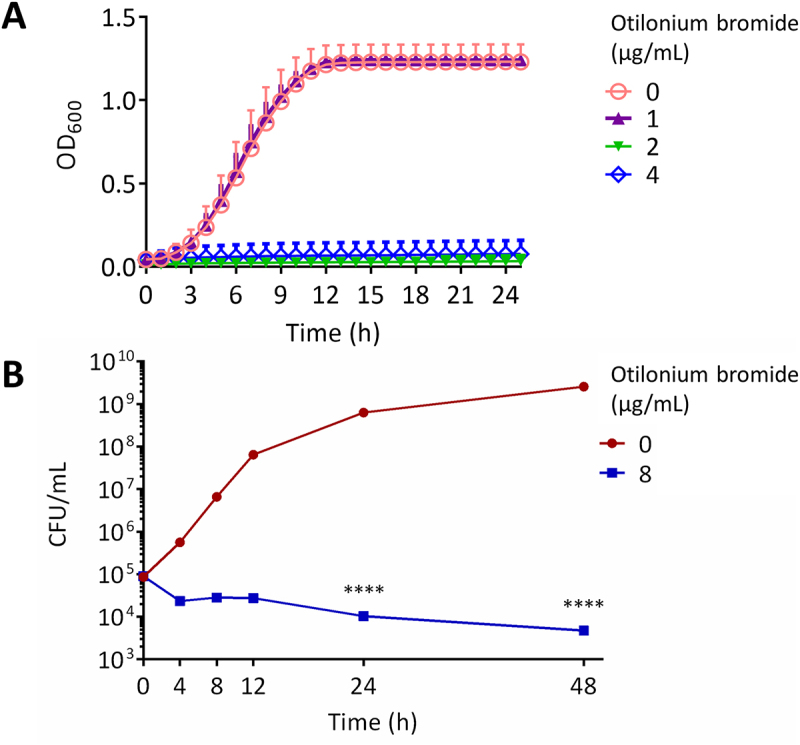
Cells of *C. albicans* were prevented from growth and were eliminated while treated with otilonium bromide.

*C. albicans* exhibited reduced growth while being treated with OB; therefore, we further tested the viability of these cells through propidium iodide (PI) staining. Consistently, PI as a membrane impermeable stain, which can distinguish live cells, could diffuse into cells treated with OB at 1 μg/mL and showed partial staining compared to untreated cells (Figure 3). On the other hand, at both 2 and 4 μg/mL of OB, *C. albicans* cells were fully stained, and remained at low optical density when measured with a spectrophotometer (Figure 4(A)). Time-kill assay revealed that *C. albicans* cells were significantly decreased after incubation for 24 h with OB, and >90% cells were eliminated after 48 h (Figure 4(B)). In brief, *C. albicans* was killed through the membrane damage induced by OB, which functions as a fungicidal compound.

*C*. *albicans* SC5314 overnight culture was washed twice with ddH_2_O, diluted with 2 mL RPMI 1640 medium to 0.25 OD_600_, and otilonium bromide was added at indicated concentrations. After 3 h incubation at 37°C, cells were washed twice with ddH_2_O, stained with 1 μg/mL propidium iodide (PI), and observed with a fluorescence microscope at 1000X. Scale bar = 5 μm.

(A). *C. albicans* overnight culture was washed twice with ddH_2_O, diluted to OD_600_ = 0.02 using 200 μL YPD broth medium with otilonium bromide at 0, 1, 2, or 4 μg/mL, and incubated at 30°C. The OD_600_ values were measured every hour. (B). *C. albicans* SC5314 overnight culture was washed twice with ddH_2_O, diluted to 10^5^ cells/mL using 3 mL YPD broth medium with otilonium bromide at 0 or 8 μg/mL, and incubated at 30°C/200 rpm. The 100 μL of culture at each time point (0, 4, 8, 12, 24, or 48 h) were properly diluted, plated on YPD agar medium, and incubated at 30°C for 48 h before colony count. **** represented *p*-value < 0.0001.

### Biofilm formation was reduced in *C. albicans* treated with otilonium bromide

**Figure 5.**
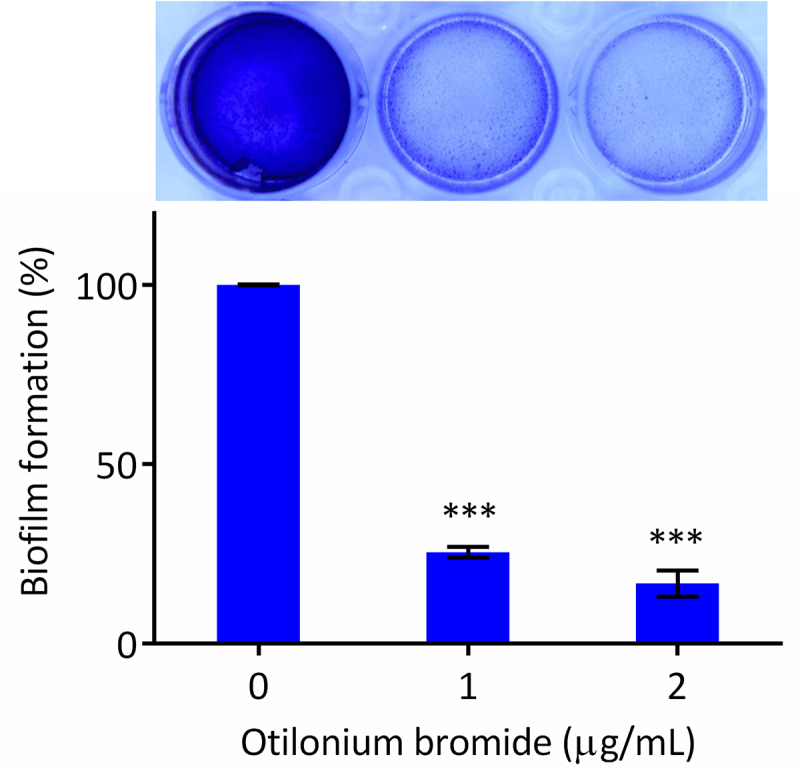
Biofilm formation was reduced in *C. albicans* treated with otilonium bromide.

Biofilm formation is also a virulence factor in *C. albicans* after its invasion and filamentation and is sometimes related to drug resistance [31]. Therefore, crystal violet was used to stain the biofilm structure to investigate how effective OB was in reducing biofilm formation in *C. albicans*. Cells adhered to the bottom of wells were allowed to form biofilm in the absence of OB. The percentage of biofilm formation decreased more than 90% and more than 95% in the presence of 2 μg/mL and 4 μg/mL OB, respectively (Figure 5). As a result, OB could significantly reduce the biofilm formation through a potential mechanism that destroyed the cell membrane and interrupted filamentation in *C. albicans*.

Overnight culture of *C. albicans* SC5314 was diluted with Spider medium to 0.5 OD_600_, and otilonium bromide was added after 1 h incubation at 37°C for cell adhesion at indicated concentrations. After 48 h incubation, biofilm was stained with crystal violet and destained with EtOH. The OD_595_ was measured after destaining, and wells without otilonium bromide were set as 100% biofilm formation. The asterisk represents significant difference (unpaired t-test, *p* < 0.05).

### RNA sequencing analysis

**Figure 6.**
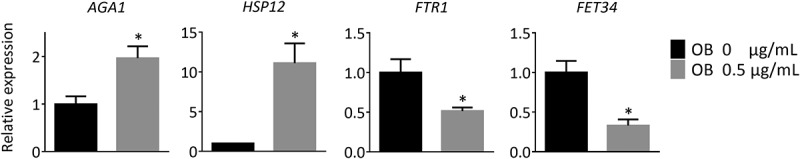
Relative gene expressions in *C. albicans* treated with otilonium bromide.

To investigate the potential targets of OB in *C. albicans*, we conducted RNA sequencing. We found 143 genes with significantly differential gene expressions (DGEs), defined as equal to or more than a 4-fold change in gene expression. Among these genes, 108 were upregulated, and 35 were downregulated in *C. albicans* treated with 0.5 μg/mL OB (Table 2). The main functions of these genes included oxidoreductase activity, heme binding, and tetrapyrrole binding, and all the functions analyzed from gene ontology indicated metal ions might play a critical role in OB-mediated gene regulation. For instance, Fet34 and Ftr1 controlled iron homeostasis in *C. albicans*, and heme binding proteins such as Rbt5 were involved in iron acquisition in the host environment; tetrapyrrole functioned as a metal ion chelator. Furthermore, heat shock proteins and cell wall-related genes were also regulated. To confirm the relative gene expression obtained from RNA sequencing, qRT-PCR experiments were conducted, and 2 genes with significant DGE were selected relatively from up- and down-regulated genes (Figure 6). All of the sequencing data were submitted to NCBI Gene Expression Omnibus (https://www.ncbi.nlm.nih.gov/geo/query/acc.cgi?acc=GSE189386, token: mfczcciyfxodteb).

Relative gene expressions were calculated through real-time qRT-PCR based on *ACT1*-calibrated and 2^−ΔΔCT^ values. The relative expression levels of *C. albicans* genes in the presence of 0.5 μg/mL otilonium bromide were normalized to those in the absence of otilonium bromide. The asterisk represents significant difference (unpaired t-test, *p* < 0.05).

**Table 2.** Otilonium bromide-mediated gene regulation.

CGDID	Gene	Description	log_2_FC
Genes upregulated in *C. albicans* treated with otilonium bromide (108/143)			
CAL0000180646	*AGA1*	Protein with similarity to agglutinin subunit	8.99
CAL0000197297	*WH11*	White-phase yeast transcript; expression in opaques	7.82
CAL0000177418	*C7_00870W*	Putative guanine deaminase	7.09
CAL0000198254	*C1_02800W*	Protein of unknown function	6.85
CAL0000177213	*PLB1*	Phospholipase B	6.44
CAL0000198834	*RBR1*	Glycosylphosphatidylinositol (GPI)-anchored cell wall protein	6.44
CAL0000188028	*CR_03320C*	hypothetical protein	6.18
CAL0000191795	*ADH3*	Putative NAD-dependent (R,R)-butanediol dehydrogenase	5.99
CAL0000179373	*CPD2*	Protein with homology to NADH dehydrogenase	5.98
CAL0000182697	*HSP12*	Heat-shock protein	5.66
CAL0000180850	*C5_02110W*	Putative heat shock protein	5.66
CAL0000176228	*C2_07630C*	Possible stress protein	5.63
CAL0000187233	*ASR1*	Heat shock protein	5.33
CAL0000189971	*RTA3*	7-transmembrane receptor protein	5.21
CAL0000175449	*CSH1*	Aldo-keto reductase	5.09
CAL0000179752	*C1_10170W*	Putative adhesin-like protein	4.84
CAL0000189325	*INO1*	Inositol-1-phosphate synthase	4.61
CAL0000175827	*C1_04010C*	Protein with a NADP-dependent oxidoreductase domain	4.49
CAL0000182669	*CSP37*	Hyphal cell wall protein	4.47
CAL0000190475	*ASR2*	Adenylyl cyclase and stress-responsive protein	4.46
CAL0000199237	*C2_02220C*	hypothetical protein	4.26
CAL0000180798	*TNA1*	Putative nicotinic acid transporter	4.01
CAL0000186516	*CDR1*	Multidrug transporter of ABC superfamily	3.76
CAL0000191119	*PDR16*	Phosphatidylinositol transfer protein	3.64
CAL0000189321	*C4_03000C*	Planktonic growth-induced gene	3.38
CAL0000194637	*C1_14060W*	Glucan 1,3-beta-glucosidase	3.34
CAL0000180820	*C2_02910W*	hypothetical protein	3.34
CAL0000197271	*C2_08890W*	hypothetical protein	3.31
CAL0000190633	*SOU1*	Enzyme involved in utilization of L-sorbose	3.31
CAL0000197522	*C3_03460C*	Protein of unknown function	3.27
CAL0000177800	*YDC1*	Alkaline dihydroceramidase; involved in sphingolipid metabolism	3.25
CAL0000176590	*DDR48*	Immunogenic stress-associated protein	3.22
CAL0000187177	*SOU2*	Protein similar to Sou1	3.22
CAL0000175241	*UGA6*	Putative GABA-specific permease	3.11
CAL0000198101	*C5_00810C*	Has domain(s) with predicted heme binding and peroxidase activity	3.10
CAL0000191057	*C1_11200W*	Predicted mucin-like protein	3.04
CAL0000197958	*ROA1*	Putative PDR-subfamily ABC transporter involved in azole sensitivity	3.04
CAL0000197123	*C7_00770W*	hypothetical protein	3.03
CAL0000191729	*ECM331*	GPI-anchored protein	3.00
CAL0000176060	*AQY1*	Aquaporin water channel	2.94
CAL0000190248	*ARG1*	Argininosuccinate synthase	2.93
CAL0000197450	*IFU5*	Predicted membrane protein involved in cell wall maintenance	2.90
CAL0000178211	*AHP1*	Alkyl hydroperoxide reductase	2.90
CAL0000190001	*C2_07440C*	Ortholog(s) have sterol esterase activity	2.86
CAL0000199842	*C6_03280W*	hypothetical protein	2.83
CAL0000184548	*CR_09140C*	Protein in directing meiotic recombination events	2.83
CAL0000174577	*OYE32*	NAD(P)H oxidoreductase family protein	2.81
CAL0000199973	*C2_06630C*	hypothetical protein	2.78
CAL0000178196	*PGA13*	GPI-anchored cell wall protein involved in cell wall synthesis	2.78
CAL0000186885	*C2_03110W*	Protein of unknown function	2.74
CAL0000180384	*YMX6*	Putative NADH dehydrogenase	2.72
CAL0000193774	*RME1*	Zinc finger protein	2.72
CAL0000189501	*TAC1*	Zn(2)-Cys(6) transcriptional activator of drug-responsive genes	2.71
CAL0000197194	*PST1*	Flavodoxin-like protein involved in oxidative stress protection	2.70
CAL0000190905	*MNN41*	Ortholog(s) have enzyme activator activity	2.69
CAL0000200522	*C7_04090C*	Predicted mitochondrial cardiolipin-specific phospholipase	2.59
CAL0000196854	*ECM4*	Cytoplasmic glutathione S-transferase	2.59
CAL0000187831	*MOH1*	Ortholog of *S. cerevisiae* Moh1, essential for stationary phase growth	2.59
CAL0000188193	*AAP1*	Putative amino acid permease	2.58
CAL0000198578	*C5_04470C*	Predicted RNA binding protein	2.56
CAL0000189861	*GRP2*	NAD(H)-linked methylglyoxal oxidoreductase	2.45
CAL0000193318	*ARE2*	Acyl CoA: sterol acyltransferase	2.43
CAL0000198473	*C1_10280C*	Putative protein of unknown function	2.40
CAL0000178438	*TRY6*	Helix-loop-helix transcription factor	2.40
CAL0000200650	*C1_05440C*	Protein of unknown function	2.39
CAL0000195719	*GRX1*	Putative glutaredoxin	2.39
CAL0000185031	*C2_10650W*	Protein of unknown function	2.37
CAL0000194072	*GLX3*	Glutathione-independent glyoxalase	2.36
CAL0000190671	*C2_06890C*	Similar to oxidoreductases	2.36
CAL0000180176	*C1_10240C*	Protein of unknown function	2.34
CAL0000183255	*C1_03870C*	Predicted heme-binding stress-related protein	2.34
CAL0000194858	*SAP4*	Secreted aspartyl proteinase	2.33
CAL0000182133	*MRF1*	Putative mitochondrial respiratory protein	2.29
CAL0000200153	*CR_08670C*	Protein with an enoyl-CoA hydratase-related domain	2.29
CAL0000191751	*ASR3*	Adenylyl cyclase and stress-responsive protein	2.28
CAL0000180926	*C3_04310C*	hypothetical protein	2.27
CAL0000201827	*PGA7*	GPI-linked hyphal surface antigen	2.27
CAL0000198851	*C7_02140W*	hypothetical protein	2.27
CAL0000190381	*C1_11950W*	Protein of unknown function	2.26
CAL0000195131	*RBT5*	GPI-linked cell wall protein	2.25
CAL0000193825	*C3_02140C*	hypothetical protein	2.24
CAL0000191735	*XKS1*	Putative xylulokinase	2.24
CAL0000201116	*CAN1*	Basic amino acid permease	2.22
CAL0000201829	*IFD6*	Aldo-keto reductase	2.21
CAL0000174172	*LCB4*	Putative sphingosine kinase	2.21
CAL0000191559	*C6_00930C*	hypothetical protein	2.18
CAL0000177288	*ARG8*	Putative acetylornithine aminotransferase	2.18
CAL0000195767	*ARA1*	D-Arabinose dehydrogenase	2.17
CAL0000179259	*C7_02920W*	Has domain(s) with predicted carbon-nitrogen ligase activity	2.16
CAL0000182975	*C4_05250W*	Putative ubiquitin-protein ligase	2.16
CAL0000181678	*C1_12140W*	Has domain(s) with predicted cofactor binding activity	2.10
CAL0000193984	*C4_02620C*	Carbohydrate kinase domain-containing protein	2.09
CAL0000201755	*TFS1*	Putative carboxypeptidase y inhibitor	2.08
CAL0000191430	*C6_03050C*	Protein of unknown function	2.08
CAL0000192412	*UGA2*	Predicted succinate semialdehyde dehydrogenase	2.08
CAL0000174960	*CIP1*	Possible oxidoreductase	2.08
CAL0000181317	*ECI1*	Protein similar to *S. cerevisiae* Eci1p	2.08
CAL0000189087	*C1_10360C*	Putative protein of unknown function	2.07
CAL0000193502	*C4_04140W*	Protein with a predicted endonuclease/exonuclease/phosphatase	2.06
CAL0000188954	*C4_02340W*	Putative protease B inhibitor	2.06
CAL0000185914	*C4_02740W*	Protein of unknown function	2.05
CAL0000196224	*C1_03150C*	hypothetical protein	2.05
CAL0000177093	*ERG2*	C-8 sterol isomerase	2.04
CAL0000192083	*OSM2*	Putative mitochondrial fumarate reductase	2.04
CAL0000178766	*C3_00210C*	Predicted integral membrane protein	2.03
CAL0000179816	*AMS1*	Putative alpha-mannosidase	2.02
CAL0000175340	*DLD1*	Putative D-lactate dehydrogenase	2.02
CAL0000177748	*C2_09590C*	Ortholog(s) have copper ion transmembrane transporter activity	2.01
Genes down-regulated in *C. albicans* treated with otilonium bromide (35/143)			
CAL0000198669	*JEN2*	Dicarboxylic acid transporter	−6.66
CAL0000188676	*SOD5*	Cu-containing superoxide dismutase	−5.31
CAL0000193948	*C1_09500W*	hypothetical protein	−5.02
CAL0000194292	*IHD1*	GPI-anchored protein	−4.92
CAL0000187820	*C6_02330W*	Described as a Gag-related protein	−4.65
CAL0000196552	*C5_02130W*	Protein of unknown function	−4.50
CAL0000180602	*TEF4*	Putative translation elongation factor	−4.08
CAL0000177231	*C1_07160C*	Protein conserved among the CTG-clade	−3.97
CAL0000181510	*NAG3*	Putative MFS transporter	−3.96
CAL0000185056	*FTR1*	High-affinity iron permease	−3.83
CAL0000192466	*FET34*	Multicopper ferroxidase	−3.75
CAL0000198258	*C3_02790W*	hypothetical protein	−3.41
CAL0000181671	*PGA26*	GPI-anchored adhesin-like protein of the cell wall	−2.99
CAL0000174751	*C1_11320C*	Protein of unknown function	−2.96
CAL0000186869	*MRV2*	Protein of unknown function	−2.95
CAL0000188660	*HAK1*	Putative potassium transporter	−2.88
CAL0000182549	*C6_01780C*	Predicted chloride transporter	−2.82
CAL0000197736	*CR_09350C*	Protein with a predicted heme oxygenase domain	−2.80
CAL0000174928	*C1_08900W*	Protein of unknown function	−2.79
CAL0000177400	*C5_03670C*	Has domain(s) with predicted metal ion binding activity	−2.59
CAL0000197551	*FCY2*	Purine-cytosine permease of pyrimidine salvage	−2.57
CAL0000193702	*C1_09210C*	Putative transporter	−2.50
CAL0000191596	*C5_03770C*	Protein similar to *Candida boidinii* formate dehydrogenase	−2.42
CAL0000180074	*FDH1*	Formate dehydrogenase	−2.42
CAL0000185898	*CFL4*	C-terminus similar to ferric reductases	−2.42
CAL0000186897	*SLP3*	Plasma membrane protein implicated in stress response	−2.40
CAL0000200616	*HGT1*	High-affinity MFS glucose transporter	−2.35
CAL0000200315	*DPP3*	Protein similar to *S. cerevisiae* pyrophosphate phosphatase Dpp1	−2.29
CAL0000198089	*GAP4*	High-affinity S-adenosylmethionine permease	−2.18
CAL0000192266	*C3_01540W*	Plasma-membrane-localized protein	−2.12
CAL0000182573	*C3_06950W*	hypothetical protein	−2.07
CAL0000190637	*FRP6*	Putative ammonia transport protein	−2.06
CAL0000198189	*C2_00760C*	Protein of unknown function	−2.06
CAL0000177585	*SEO1*	Protein with similarity to permeases	−2.05
CAL0000179202	*YHB1*	Nitric oxide dioxygenase	−2.01

### Iron acquisition was interrupted by otilonium bromide in *C. albicans*

**Figure 7.**
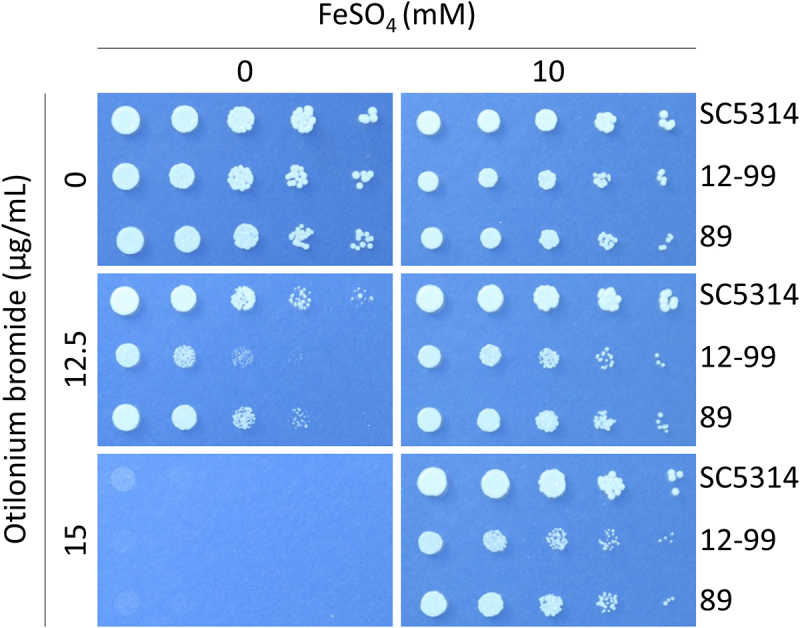
The inhibition of otilonium bromide against *C. albicans* was interrupted by the addition of FeSO_4_.

According to our present research, quaternary ammonium compounds (QACs) could inhibit *C. albicans* through a pathway like nutrient immunity in humans [7]. Based on this result, combined with the RNA sequencing data, we inferred that OB, also a QAC, would affect iron homeostasis in *C. albicans*. Spot plating assay on YNB agar medium showed that colonies of *C. albicans* hardly grew, but recovered after the addition of iron, suggesting that sufficient iron was required for *C. albicans* to conquer the fungicidal activity of OB (Figure 7).

Overnight culture of *C. albicans* SC5314, and two drug-resistant *C. albicans* 12–99 and 89 isolates were washed twice and adjusted to OD_600_ = 0.2 with ddH_2_O. Three microliters of 5-fold serial diluted cell suspensions were dropped onto YNB agar plates with or without otilonium bromide or FeSO_4_ at the indicated concentrations. Plates were incubated at 30°C for 48 h before being photographed.

### Otilonium bromide exhibited marginal therapeutic efficacy in the murine model of systemic infection

**Figure 8.**
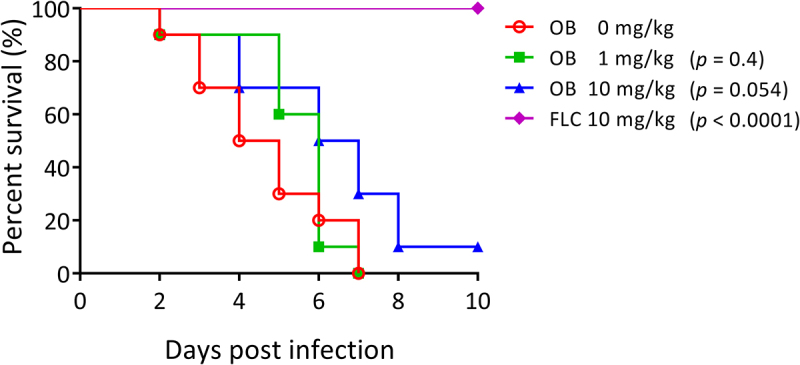
Otilonium bromide exhibited marginal therapeutic efficacy in murine model of systemic candidiasis.

A therapeutic assay in a murine model was conducted to investigate the efficacy of OB. Mice treated with 0 or 1 mg/kg OB had all died by the 7th day after *C. albicans* inoculation, while 10% those treated with 10 mg/kg still survived at the endpoint of the experiment (*p* = 0.054, Figure 8). This result represented a marginal therapeutic effect for OB in curing *C. albicans* infected mice, and this effect was likely dose-dependent.

Cells of *C. albicans* SC5314 were grown overnight in YPD medium, washed twice with PBS buffer, and adjusted to a concentration of 5 × 10^6^ cells/mL with PBS buffer. Mice were inoculated with 10^6^ *C. albicans* cells in 200 μL by tail-vein injection, and the oral administration of otilonium bromide (OB) or fluconazole (FLC) at indicated concentrations were conducted at 4, 24, 48, 72 h after the inoculation. Ten mice per group were used, and all mice were monitored for 10 days, and data were statistically analyzed by log-rank test.

## Discussion

It was recently reported that OB can inhibit the germination of *C. neoformans*, and also possesses an inhibitory effect against *A. fumigatus* and *C. albicans* [4,32]. Based on this, we found that OB not only prevented *C. albicans* from yeast-to-hyphae transition but also killed yeast or early germinated cells, indicating the novel antifungal activity of this drug. OB has been used to treat irritable bowel syndrome (IBS). Interestingly, an increased amount of *C. albicans* could be found in IBS patients [33,34], suggesting fungal dysbiosis may lead to gastrointestinal dysfunctions, and the disease might be cured with OB after future development.

Yeast-to-hyphae transition is an important virulence factor in *C. albicans*, and it could colonize and form biofilm in the kidney, spleen or liver to defend against foreign compounds, including antifungal drugs during infection [35]. This study found that OB inhibited *C. albicans* from such transition in a dose-dependent manner and considerably reduced biofilm formation, indicating that the virulence and defensive ability of *C. albicans* against antifungal drugs may decrease. Nevertheless, in our therapeutic assay, OB showed marginal therapeutic efficacy in a murine model of systemic infection. Since OB suppresses IBS by blocking Ca^2+^ channels or tachykinin receptors on nerve cells of the intestinal wall, it could be inferred that there might be a lack of sufficient OB to inhibit pathogens in the kidneys, spleens or liver, in which *C. albicans* usually colonized [9]. Thus, we speculate that providing a higher dose of OB may increase the therapeutic efficacy. However, there was no significant therapeutic effect while mice were treated with a higher dosage (20 mg/kg) as well as intermediate doses (2 and 5 mg/kg) (Fig. S1). Meanwhile, further chemical modifications of the OB molecule may enhance therapeutic activity against *C. albicans*. Indeed, our recent study showed that quaternization with N-butylsulfonate may improve antifungal properties of the OB derivatives, and the incorporation of a 3-aminobenzoic acid moiety had a favorable impact on the compounds, further enhancing their antifungal efficacy. However, these derivatives did not display an MIC against *C. albicans* lower than that in OB with original structure [36].

QACs were found to have broad-spectrum antimicrobial activity, due to their activity on the bacterial cell membrane [37,38]. In our previous study, we found that another QAC, PMT12-BF4, also inhibited fungal pathogens such as *C. albicans*, non-*albicans Candida* species and *A. fumigatus* [7]. Similarly, the inhibitory effect of OB was abolished by replenishing iron ions. RNA sequencing data showed that many transcripts associated with iron homeostasis were expressed differentially in *C. albicans* treated with OB, indicating that OB might inhibit *C. albicans* through destabilizing iron homeostasis. Iron deficiency also mediated cell wall composition by, for example, decreasing β-1, 3-glucan in *C. albicans*. Under high iron conditions, *C. albicans* was able to resist cell wall-perturbing antifungals [39]. Compared to our spot plating assay, the non-sufficient iron conditions caused by OB might indirectly decrease the cell wall integrity of *C. albicans*, resulting in growth reduction.

The relationship between calcium signaling and iron homeostasis in fungal pathogens has recently emerged as an important area in antifungal research. While OB is known to affect calcium channels in mammalian cells [9], our study revealed its impact on iron homeostasis in *C. albicans*. Recent research has shown that calcium and iron homeostasis are intricately linked in fungal pathogens. In brief, iron deficiency may lead to calcium overload, while calcium supplementation could suppress iron uptake-related gene expression [40]. Specifically, under iron-deficient conditions, cells may mistakenly absorb calcium as a substitute, leading to disturbed ion homeostasis and growth inhibition. This mechanism was particularly relevant to our findings with OB, where we observed that iron supplementation could rescue the growth inhibition effect. The interplay between calcium and iron might contribute to OB’s antifungal mechanism in several ways. The iron deficiency caused by OB might lead to calcium overload and increased intracellular reactive oxygen species (ROS) production, further contributing to its antifungal activity. This dual effect on both calcium and iron homeostasis could explain efficacy of OB against *C. albicans* and suggested potential advantages in combating drug resistance.

In recent study, iron chelator was reported to synergize with antifungal drugs. The fluconazole-resistant *C. albicans* strain was found enhancing iron uptake to maintain its resistance; however, it was more sensitive to fluconazole while in hosts treated with iron chelators, suggesting that iron availability played an important role in azole resistance [41]. Compared to our results, OB may cause iron depletion, which was similar to the function of iron chelators, and thus disrupted iron availability of *C. albicans*, resulting in growth defect. In fact, iron chelators could also be antifungal agents that inhibit *C. albicans* from growth [42–44].

Calcineurin is a calcium and calmodulin-dependent serine/threonine protein phosphatase that plays a crucial role in immune responses [45]. Although OB and calcineurin both play roles in modulating calcium signaling pathways, they affect different tissues and systems. While calcineurin activity is dependent on calcium signaling in immune cells, OB acts by inhibiting calcium channels in smooth muscle cells. Results showed that OB could also inhibit *C. albicans* calcineurin mutants *Δcna1/Δcna1* and *Δcnb1/Δcnb1* (Table 1), suggesting specific pathways regulated by calcineurin might not participate in the antifungal mechanism of OB. However, whether there could be pharmacological or physiological interactions if OB and calcineurin inhibitors are used in a clinical setting is not clear, and how calcium dynamics simultaneously affect both smooth muscle contraction and immune responses also needs to be clarified. If OB acts independently, it suggests broader potential for combination therapy or effectiveness.

In conclusion, this study demonstrated that OB, an FDA-approved drug for IBS treatment, exhibited promising antifungal activity against *C. albicans in vitro* through interference with iron homeostasis. OB effectively inhibited both yeast-to-hyphae transition and biofilm formation, which were two critical virulence factors of *C. albicans*. While its *in vivo* efficacy was limited, possibly due to distribution challenges and complex host environments, our findings provided valuable insights for future antifungal drug development. The established safety profile of OB, combined with its novel antifungal mechanism, suggesting that chemical modifications or alternative delivery strategies could potentially enhance its therapeutic efficacy against fungal infections. Further investigation of iron homeostasis as a target for antifungal drug development may also lead to new therapeutic approaches.

## Supplementary Material

ARRIVE guidelines checklist 20250902.pdf

Hsu et al_Supplementary_20250609.docx

## Data Availability

The authors confirm that the data supporting the findings of this study are available within the article or its supplementary materials. The data that support the findings of this study are openly available in figshare at http://dx.doi.org/10.6084/m9.figshare.27434217 [46].

## Data collection

The data collection process was conducted between 9 October 2018 and 22 August 2024.

Main experiments were performed during the period between 9 October 2018 and 11 May 2021. Data organization, validation, and statistical analysis were carried out from 30 August 2019 to 28 October 2021. Data gathering and analyzing were conducted from 3 April 2024 to 22 August 2024.
