# Supplementary material for: Otilonium bromide exhibits novel antifungal activity against Candida albicans via regulating iron homeostasis
Source: Virulence. 2025 Dec 23;17(1):2609407. doi: 10.1080/21505594.2025.2609407 (PMC12758266; doi:10.1080/21505594.2025.2609407)
Supplement: Hsu et al_Supplementary_20250609.docx [file KVIR_A_2609407_SM7354.docx]

**Supplementary table S1**. Primer pairs used in real-time qRT-PCR to confirm RNA sequencing results

| **Gene** | **Primer pair sequences** |
| --- | --- |
| *AGA1* | GCCCCAATGGAACCACTGTTAT |
|  | AGCCTGTTGTTGCTGTGGTTGT |
| *HSP12* | TACCTTGGAAAAGGGCAAGGAA |
|  | TGCACGTTGTCTGCAACAGTTT |
| *FTR1* | TAATTGCCGGTATCGTTGTTGG |
|  | CCGTTTTCAGAAGCATCACCAC |
| *FET34* | TTGGCCAGAATATCCAATGGTG |
|  | ATGGCAGTGGAAGAACCAAACA |





**Supplementary figure S1.** Survival curves of mice treated with **(A)** 0, 2, and 5 mg/kg or with **(B)** 0 and 20 mg/kg otilonium bromide (OB). Fluconazole (FLC) in a concentration of 10 mg/kg represented a positive control.

Cells of *C. albicans* SC5314 were grown overnight in YPD medium, washed twice with PBS buffer, and adjusted to a concentration of 5 x 10^6^ cells/mL with PBS buffer. Mice were inoculated with 10^6^ *C. albicans* cells in 200 μL by tail-vein injection, and the oral administration of OB or FLC at indicated concentrations were conducted at 4, 24, 48, 72h after the inoculation.
